# Unravelling Impaired Hypoalgesia at Rest and in Response to Exercise in Patients with Chronic Whiplash-Associated Disorders: Effects of a Single Administration of Selective Serotonin Reuptake Inhibitor versus Selective Norepinephrine Reuptake Inhibitor

**DOI:** 10.3390/jcm12154977

**Published:** 2023-07-28

**Authors:** Margot De Kooning, Iris Coppieters, Eva Huysmans, Jo Nijs, Mira Meeus, Lennard Voogt, Erwin Hendriks, Kelly Ickmans

**Affiliations:** 1 Pain in Motion Research Group (PAIN), Department of Physiotherapy, Human Physiology and Anatomy, Faculty of Physical Education & Physiotherapy, Vrije Universiteit Brussel, 1090 Brussels, Belgium; margot.de.kooning@vub.be (M.D.K.); iris.coppieters@vub.be (I.C.); eva.huysmans@vub.be (E.H.); l.p.voogt@hr.nl (L.V.); erwin.paul.hendriks@vub.be (E.H.); kelly.ickmans@vub.be (K.I.); 2 Department of Physical Medicine and Physiotherapy, University Hospital Brussels, 1020 Brussels, Belgium; 3 Pain in Motion International Research Group, 1090 Brussels, Belgium; mira.meeus@uantwerpen.be; 4 Unit of Physiotherapy, Department of Health and Rehabilitation, Institute of Neuroscience and Physiology, Sahlgrenska Academy, University of Gothenburg, 405 30 Göteborg, Sweden; 5 Department of Public Health (GEWE), Faculty of Medicine and Pharmacy, Vrije Universiteit Brussel, 1090 Brussels, Belgium; 6 Research Foundation Flanders (FWO), 1000 Brussels, Belgium; 7 Movant, Department of Rehabilitation Sciences and Physiotherapy, Faculty of Medicine and Health Sciences, University of Antwerp, 2000 Antwerpen, Belgium; 8 Research Centre for Health Care Innovations, Rotterdam University of Applied Sciences, 3015 GG Rotterdam, The Netherlands; 9 Rehabilitation Centre Drechtsteden/Haaglanden, Berkenhof 100, 3319 WB Dordrecht, The Netherlands; 10 Unit Physiotherapy, Organizational Part of the Orthopedics Department, Erasmus Medical Centre, 3015 GD Rotterdam, The Netherlands

**Keywords:** chronic whiplash-associated disorders, serotonin, noradrenaline, exercise, pain, exercise-induced hypoalgesia

## Abstract

(1) Background: Noradrenaline and serotonin have modulatory roles in pain signaling and in exercise-induced hypoalgesia. Patients with chronic whiplash-associated disorders often show impaired exercise-induced hypoalgesia. Therefore, this study aimed to examine the isolated effect of activating serotonergic or noradrenergic descending pathways on hypoalgesia at rest and in response to exercise in patients with chronic WAD by using respectively a single dose of a selective serotonin reuptake inhibitor (SSRI) and a selective norepinephrine reuptake inhibitor (NRI). (2) Methods: Twenty-five people with chronic WAD participated in this double-blind randomized controlled crossover experiment. Serotonin and noradrenaline concentrations were modulated by the oral ingestion of a single dose of citalopram (i.e., SSRI) or atomoxetine (i.e., SNRI). Quantitative sensory testing (including pressure pain thresholds and conditioned pain modulation) was measured before and after exercise in combination with no medication (1), atomoxetine (2), or citalopram (3) at three different test days. (3) Results: Random-intercept linear mixed models analysis was used to analyze pain outcomes (i.e., pain at rest and exercise-induced hypoalgesia) before and after exercise over the three conditions in patients with chronic WAD. No differences in pain at rest were found between the three conditions before exercise. The effect of exercise on pain outcome measures was not influenced by medication intake. The occupational status of the participants had a significant influence on the effect of exercise and medication on pain outcomes (*p* < 0.05). Patients working full-time had some positive effect of atomoxetine on pain facilitation (*p* < 0.05). Unemployed patients had some negative effect of citalopram on pain tolerance and experienced exercise-induced hypoalgesia (*p* < 0.05). (4) Conclusions: A single dose of citalopram or atomoxetine did not result in changes in hypoalgesia at rest and in response to exercise. These results do not support the use of SSRI or selective NRI to overcome impaired hypoalgesia at rest or in response to exercise in people with chronic WAD. Effect of exercise and medication on pain in patients with chronic WAD is influenced by the occupational status.

## 1. Introduction

The monoamines noradrenaline and serotonin have modulatory roles in pain signaling [1]. Descending pain modulatory pathways arise from the midbrain and brainstem and exert control over the dorsal horn neuronal responses to noxious stimulation. This powerful modulation can be both inhibitory or excitatory and is mainly the result of the neurotransmitters’ noradrenaline and serotine (5-hydroxytryptamine, 5-HT) effect on specific receptor subtypes [2]. While noradrenaline exerts an inhibitory effect mainly through α2-adrenoceptor activation, serotonin has a bi-directional role in pain processing, which includes an inhibitory action by 5-HT_7_ receptor activation and pain facilitation through 5-HT_3_ receptor activation [1,3]. This descending inhibitory control results in the reduction of transmitted signals, which can further lead to hypoalgesia. Centrally acting medication such as antidepressants have the opportunity to alter serotonin and noradrenaline activity by inhibiting the reuptake of these monoamines into the presynaptic neuron. Evidence supports the hypoalgesic effects of centrally acting medication in people with chronic pain [4]. These effects are specifically well studied in trials with serotonin-noradrenaline reuptake inhibitors, known as SNRIs [4]. However, less research data are available regarding the isolated hypoalgesic effect of selective serotonin reuptake inhibitors (SSRIs) and selective noradrenaline reuptake inhibitors (NRIs) in patients with chronic pain. Unravelling the contribution of serotonin and noradrenaline separately in the descending pain control pathways and their hypoalgesic effects is of clinical relevance for people suffering from chronic pain conditions. Indeed, these centrally acting reuptake inhibitors can be a useful treatment for patients with chronic pain conditions [5] such as chronic whiplash-associated disorders (WAD).

WAD are the result of an acceleration–deceleration trauma of the neck. Patients with chronic WAD primarily have persistent neck pain, but the clinical representations can also include fatigue, concentration difficulties, headaches, and reduced quality of life [6,7]. The chronic pain experience in chronic WAD might be the result of plastic changes in the central nervous system causing altered central pain processing or central sensitization [8]. Central sensitization comprises generalized hypersensitivity to a variety of stimuli caused by an amplification of neural signaling in the central nervous system [9]. Features of central sensitization includes impaired hypoalgesia at rest and in response to exercise (known as “exercise-induced hypoalgesia”) [10,11]. Patients with chronic WAD demonstrate impaired hypoalgesia at rest [12] and in response to exercise [13]. 

In normal circumstances, aerobic exercise will lead to an increase in pain thresholds and a decrease in pain ratings [14,15]. This phenomenon is called exercise-induced hypoalgesia and is caused by the activation of opioidergic, spinal, and supraspinal inhibitory mechanisms [1]. In both human and animal studies, it has been demonstrated that serotonin is involved in exercise-induced hypoalgesia [16]. Exercise induces an increase in serotonin concentrations in the brain, including brain areas that are involved in pain control [17]. Besides the involvement of this serotonin system, a noradrenaline system is involved in exercise-induced hypoalgesia as well. Nonetheless, fewer studies have investigated its involvement, and the majority were performed on rodents [16]. Investigating the possible role of serotonin and noradrenaline systems in exercise-induced hypoalgesia is of specific interest to patients with impaired exercise-induced hypoalgesia, such as people with chronic WAD [13,18]. SSRIs and selective NRIs alter neurotransmitter concentrations and hence may possibly fortify the hypoalgesic effect during exercise. Studies examining the role of serotonin and adrenaline in hypoalgesia at rest and in response to exercise in patients with chronic WAD are essentially lacking and represent an important knowledge gap. Studying the role of serotonin and adrenaline in hypoalgesia at rest and in response to exercise can improve the knowledge of the underlying mechanisms as well as open new perspectives for clinical use of SSRI and selective NRI in people with chronic WAD. 

For the reasons outlined above, this study aims at unravelling the biological nature of impaired hypoalgesia at rest and in response to exercise in patients with chronic WAD. More specifically, it is aimed to examine the isolated effect of activating serotonergic or noradrenergic descending pathways on hypoalgesia at rest and in response to exercise in patients with chronic WAD by using respectively a single dose of a selective serotonin reuptake inhibitor (SSRI) and a selective norepinephrine reuptake inhibitor (NRI). We hypothesized that activation of serotonergic and/or noradrenergic descending pathways would improve hypoalgesia at rest and in response to exercise in patients with chronic WAD. In addition, it is aimed at examining whether activation of serotonergic and/or noradrenergic descending pathways prevents post-exertional malaise following submaximal exercise in chronic WAD patients. 

## 2. Materials and Methods

### 2.1. Study Design and Setting

**Figure 1:**
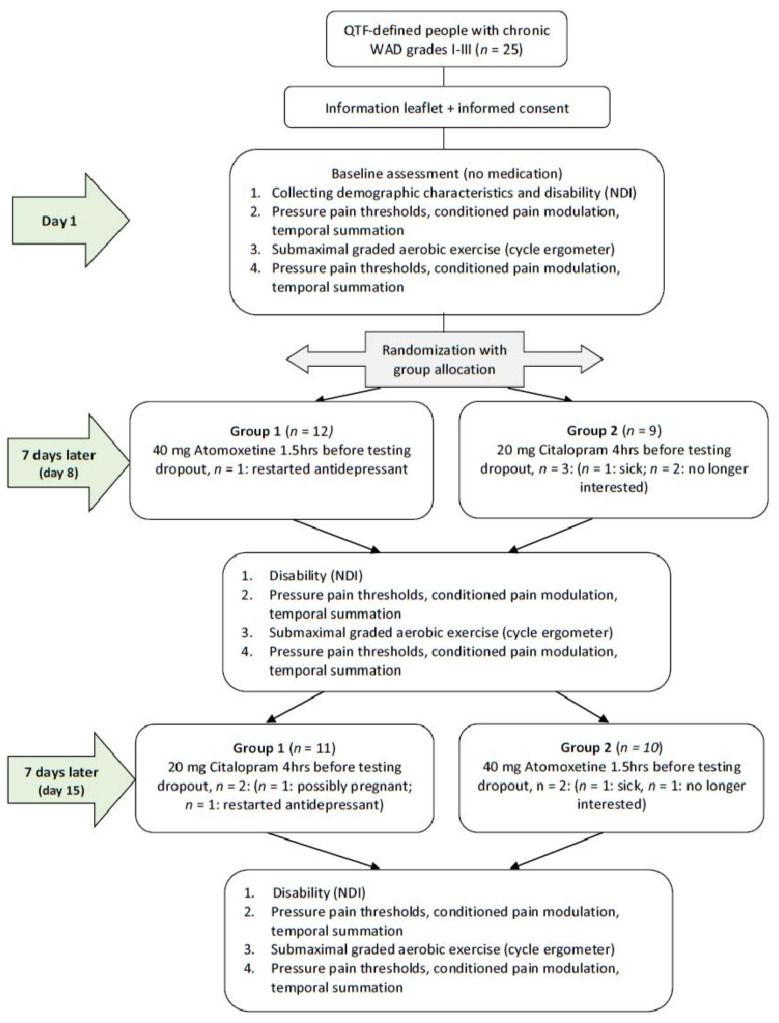
Flowchart of the study. QTF = Quebec Task Force; WAD = whiplash-associated disorders; NDI = Neck Disability Index.

In order to examine the study aims, the study comprised a double-blind randomized controlled crossover experiment comparing three conditions: (1) no medication intake (baseline condition), (2) after intake of 20 mg citalopram (SSRI), and (3) after intake of 40 mg atomoxetine (selective NRI). Figure 1 presents a flowchart of the study design. The study took place at the Department of Physical Medicine and Physiotherapy of the University Hospital Brussels (Brussels, Belgium). The research protocol was approved by the Ethics Committee of the University Hospital Brussels/Vrije Universiteit Brussel. All participants were thoroughly informed about the study procedures and signed an informed consent prior to study enrolment. The study was registered at ClinicalTrials.gov (Identifier No. NCT01601912). 

The a priori sample size calculation was conducted with G*Power 3.1.5 (Kiel, Germany) [19]. The sample size calculation for the within-group analysis (pre vs. post exercise) is based on the study results of our past study revealing impaired exercise-induced hypoalgesia in patients with chronic WAD [13]. The sample size was calculated using a desired power of 1 − β = 0.80, a significance level of α = 0.05, and an effect size of d = 0.30. Calculation was based on the standard deviation. Based on this a priori sample size calculation, we aimed at enrolling sufficient participants with chronic WAD to have at least 15 participants after dropout. Following the advice of Hoenig and Heisey, no post hoc power calculation was executed to prevent interpretations made on flawed reasoning [20].

### 2.2. Participants

In order to prevent recruitment bias and optimize the external validity of the study findings [21], people with chronic WAD were recruited through various ways, including the departments of physical medicine, physiotherapy, and emergency medicine of the University Hospital Brussels, a peripheral center for emergency medicine and rehabilitation, as well as through social media and advertisements placed in the newsletter and on the website of a Belgian patient-support group (i.e., the non-profit organization vzw Whiplash). 

People with chronic WAD were eligible if they had persistent neck pain lasting at least 3 months resulting from a motor vehicle collision or traumatic event classifiable as WAD I, II, or III according to the Quebec Task Force criteria [7]. Furthermore, all participants were native Dutch speakers and between 18 to 65 years old. Exclusion criteria included (1) initial fulfilment of the WAD grade IV Quebec Task Force criteria (i.e., fracture or dislocation of the cervical spine) [7]; (2) being pregnant or ≤ 1 year postnatal; (3) intellectual disabilities; and (4) other past and/or current comorbidities or health issues that could explain the pain complaints. Furthermore, included patients were (if applicable) instructed to stop the use of opioid analgesics, antidepressants, and anti-epileptic medication 2 weeks prior to study participation. On each assessment day, the participants were asked to refrain from taking non-opioid analgesics and beta-adrenergic blocking agents; not to consume caffeine, alcohol, and nicotine; and not to undertake physical exertion. For ethical reasons, patients were able to take non-opioid pain medication (NSAIDs, acetylsalicylic acid, and paracetamol) during the 3 weeks study period but not on the days of the assessments.

### 2.3. Procedure

Baseline assessments were performed on the first test day without medication intake. These assessments consisted of the completion of a demographic questionnaire, the Neck Disability Index (NDI), and a visual analogue scale (VAS) to investigate self-reported levels of pain. Next, pressure pain thresholds (PPTs), conditioned pain modulation (CPM), and temporal summation (TS) were examined. Pain measurements (VAS pain, PPTs, CPM, and TS) were assessed immediately before and immediately after a single submaximal aerobic exercise bout. In addition, participants were also asked to indicate their level of pain (VAS pain) 24 h after the exercise. Finally, cognitive performance was also assessed at baseline and after exercise, but the methods and results of the cognitive performance tests are reported elsewhere [22]. 

After baseline assessment, an independent researcher who was not involved in recruitment, assessments, interventions, or statistical analyses randomly allocated the participants to one of the 2 groups (group 1 = day 8: selective NRI + day 15: SSRI and group 2 = day 8: SSRI + day 15: selective NRI) according to a computer-generated randomization list. On day 1, participants received their first single dose of medication (blinded) for day 8 as well as further verbal and written instructions regarding its administration (see Section 2.4). One week after the baseline assessments, all participants were invited to visit the university hospital for the second time (day 8). Except for filling out the demographic questionnaire, the same assessments were performed as on day 1. After finishing the assessments on day 8, they received their second single dose of medication (blinded) for day 15 as well as further verbal and written instructions regarding its administration (see Section 2.4). One week after the second assessment, participants visited the university for the third time (day 15) and exactly the same assessments as on day 8 were performed. 

All assessments in each patient were performed by the same researcher. After the final assessment, the success of patient and assessor blinding was examined by asking the patient and the assessor to indicate group allocation, including the percentage of certainty (i.e., 50% certainty means a pure guess). If applicable, the reasons for withdrawal from the study were monitored. 

The measurements of a participant sample presenting considerable overlap with the current study sample was used in our previous studies with regard to the effect of exercise on pain and additional influence of SSRI and selective NRI medication on cognitive performance in people with chronic WAD and healthy controls [23,24].

### 2.4. Medication Administration

In order to ensure peak concentrations at the time of testing, all participants were instructed to take citalopram (20 mg per os; Citalopram Sandoz^®^ (Mumbai, India)) 4 h and atomoxetine (40 mg per os; Strattera^®^ (Hampshire, UK)) 1.5 h before the scheduled start of their next appointment. Participants were instructed not to ingest food thirty minutes before and thirty minutes after medication intake to ensure that the attainment of the peak concentration was not slowed down. Both pills had exactly the same visual appearance. They were transparent hard-shelled capsules filled with dry white powder.

Citalopram was chosen as SSRI due to the serotonin specificity. Citalopram activates serotonergic descending pathways [25] and acts on serotonergic neurons in the nervous system. This SSRI potentiates serotonergic transmission by selectively blocking serotonin reuptake [26]. The action of citalopram is highly selective, with minimal effects on dopamine and noradrenaline reuptake [27].

Atomoxetine is a potent noradrenalin uptake inhibitor in humans and animals, and studies have shown that it preferentially binds to areas of known high distribution of noradrenergic neurons, such as the prefrontal cortex [28]. It has a high affinity for the noradrenaline transporter and much lower affinity for the dopamine and serotonin transporters [29].

The half-life of citalopram is approximately 1 ½ day and the half-life of atomoxetine after oral administration is 3.6 h in rapid metabolizers and 21 h in slow metabolizers. The washout period of seven days is sufficient to prevent carry-over effects.

### 2.5. Submaximal Aerobic Exercise

The acute submaximal graded aerobic exercise was performed on a cycle ergometer (kardiomed 520 basic cycle; Proxomed, Alzenau, Germany), with the seat adjusted to be appropriate for each participant. Once the participant was adjusted to the resting position for 2 min and the heart rate monitor (Polar Electro Oy, Kempele, Finland) was switched on, resting heart rate was recorded. The workload started at 25 W and was increased by 25 W every minute until the participant reached the submaximal level (i.e., target heart rate). The target heart rate is defined as 75% of the age-predicted maximal heart rate: (220–age) × 0.75. Participants were instructed to cycle at a constant pedaling rate of approximately 60 rounds per minute. Heart rate was recorded at the end of every minute. The submaximal exercise was terminated when participants reached their individual target heart rate. Cooling down comprised 1 min of cycling at a workload of 25 W and a rate of 60 rounds per minute. The test duration was maintained below 15 min to avoid early fatigue in the lower extremities due to insufficient physical fitness. This exercise protocol is defined as the aerobic power index test [30], which has been demonstrated to be reliable as a submaximal exercise protocol in people with chronic pain [31].

### 2.6. Demographic Characteristics 

Demographic characteristics such as age, sex, height, weight, disease duration, occupational status, educational level, possible legal conflict, and medication use were questioned.

### 2.7. Self-Reported Measures

#### 2.7.1. Neck-Pain-Related Disability

The NDI was used to investigate neck-pain-related disability levels (0–100) [32,33]. Higher scores on the NDI indicate higher levels of pain-related disability. The NDI Dutch language version is proven to be valid and reliable within people having chronic neck pain [34,35]. 

#### 2.7.2. Self-Reported Pain Intensity

Participants had to indicate their present levels of pain on a VAS by drawing a vertical line on a 100 mm horizontal line with the left marked “no pain” and at the right “worst possible pain”. A score ranging from 0 to 100 was obtained. The VAS was filled out 3 times for each test day: immediately before, immediately after, and 24 h after the submaximal exercise to investigate post-exertional symptom changes. This patient-reported measure shows good validity and reliability [36,37].

#### 2.7.3. Experimental Pain Measurements–Quantitative Sensory Testing 

To investigate pain sensitivity, endogenous hypoalgesia, and pain facilitation, participants were subjected to experimental pain assessments: PPTs, CPM, and TS. Experimental pain measurements were executed before and after every submaximal aerobic exercise. The experimental pain assessments consisted of the following four-phase procedure. The first phase comprised the determination of the PPTs at the right shoulder (at the trapezius muscle belly in the middle between processus spinosus of C7 and lateral border of the acromion = local site) and at the right calf muscle belly (in the middle of the calf’s proximal 1/3 = remote site) with an analogue Fisher algometer (Force Dial model FDK 40 Push Pull Force Gage, Wagner Instruments, P.O.B. 1217, Greenwich CT 06836). In order to determine PPTs at each location, pressure was gradually increased at a rate of 1 kg/s until the participant reported first onset of pain (participants were instructed to say “stop” at that point). The PPT was determined as the mean of two consecutive measurements (30 s in between). 

The second phase comprised the examination of TS: at each location (i.e., right shoulder and right calf), TS was provoked by means of ten consecutive pressure pulses (test stimulus). The magnitude of these pressure pulses corresponded to the previously determined PPT (explained in the previous paragraph). To preclude bias originating from possible sensitization of PPT assessment, TS was initiated 2 min after the determination of the PPT. For each pressure pulse, pressure was gradually increased at a rate of 2 kg/s to the predetermined PPT and maintained to that point for 1 s before being released. An interstimulus interval of 1 s was used. The participants were asked to rate the intensity and unpleasantness of the pain of the 1st, 5th, and 10th pressure pulse on a verbal numerical rating scale (VNRS, 0 = no pain to 10 = worst possible pain). After TS was measured on the shoulder as well as on the calf, a rest period of 5 min was allowed before investigating CPM (i.e., phase 3 of the experimental pain assessments). TS was calculated as the VNRS score of the 10th pressure pulse minus the VNRS score of the 1st pressure pulse. 

During the third phase, the measurement of CPM was performed. The conditioning stimulus was the inflation of an occlusion cuff at the participant’s left arm to a painful intensity. The occlusion cuff was inflated at a rate of 20 mmHg/s until “the first sensation of pain” was reported. This cuff inflation was maintained for 30 s. The participant was then asked to rate the pain intensity on a VNRS (0 = no pain to 10 = worst possible pain). Depending on the VNRS score, the cuff inflation was then increased or decreased until pain intensity was rated by the participant as 3/10 (i.e., VNRS 3). Next, the above-mentioned TS procedure (i.e., phase 2) was repeated during maintenance of the cuff inflation [38,39]. To follow the consensus recommendations, CPM was calculated as the first VNRS score during cuff inflation minus the first VNRS score before cuff inflation [40]. This means that a positive value represents pain facilitation and negative values represents pain inhibition. This test protocol was found to be reliable and valid for examining CPM [38,39]. 

In sum, outcomes for the experimental pain measurements were as follows: PPT shoulder, PPT calf, TS shoulder, TS calf, CPM shoulder, CPM calf, and cuff pressure VNRS 3. 

### 2.8. Statistical Analysis

All data were analyzed using SPSS, version 25 (SPSS Inc. Headquarters, Chicago, IL, USA). Normality of demographic variables was checked using the Shapiro–Wilk test, and appropriate statistics were calculated for the participant characteristics. To assess the difference in pain outcomes between the three experimental conditions in response to the exercise intervention, a random-intercept linear mixed models analysis was applied. For each outcome measure, the best fitted model was achieved by use of the Akaike information criterion (AIC) (to estimate the relative quality of statistical models for a given set of data). Every model included the “experimental condition” (no-medication, citalopram, and atomoxetine), “time” (i.e., pre aerobic exercise, post aerobic exercise, and 24 h post-exercise in the case of VAS pain), and “experimental condition × time interaction” as fixed effects together with a random intercept for each patient. Further covariates were added in a stepwise manner and included when this led to a significant decrease in AIC. Covariates that could be included were age, sex, body mass index, duration of illness, involved in legal conflict, occupational status, use of pain medication prior to study participation, and randomization. Medication use was used to account for possible influences of medication use prior to study participation. The randomization was used as a covariate to control for possible carry-over effects and the influence of order in which medications had been administered. Neither medication nor randomization had a significant influence on the outcome measures. The covariate occupational status had a significant influence on almost all outcome parameters. Therefore, sub-analyses using random-intercept linear mixed models were carried out to investigate the main outcome parameters in the subgroups “working full-time” and “unemployed”. These two groups were formed out of the main analysis, which demonstrated a significant difference between these groups. Post hoc analyses with Bonferroni correction were executed if the factors “experimental condition”, “time”, or “experimental condition x time” appeared significant. This mixed model analysis allowed us to take dropouts into account. All available data were included in the models, even if only data of one or two of the three test days was available. *p* < 0.05 (2-sided) was considered significant.

## 3. Results

### 3.1. Group Characteristics and Self-Reported Measures

In total, 25 people with chronic WAD were included in the study (10 men and 15 women). The mean age of the participants was 40 years, ranging from 18 to 59 years. One person was using antidepressants but discontinued its use two weeks prior to baseline. Eight participants reported the use of analgesics but refrained from intake on assessment days. At baseline, the mean pain-related disability level as measured with the NDI was 40.2/100, which corresponds to a moderate level of impairment [33]. The levels of pain-related disability did not differ between assessment days (*p* > 0.05). Further characteristics of the participants are presented in Table 1. There was a 100% success of assessor blinding and 96% success of patient blinding. All 25 participants completed the assessments at baseline. However, six patients did not complete the entire study, resulting in five dropouts during the citalopram test day and three during the atomoxetine test day. This corresponds to a dropout rate of 18%. Two patients terminated the study after the baseline measurements—one due to restart of a tricyclic antidepressant, the other patient because of a long travel distance to the test facility. One patient did not participate during the atomoxetine test day due to illness. Three patients did not participate during the citalopram test day. The first patient felt ill after the intake of citalopram, the second patient was possibly pregnant, and the third patient did not give a reason for the withdrawal. The multilevel analysis allowed us to counteract loss of data due to dropout.

### 3.2. The Isolated Effect of a Single Dose of a SSRI or a Selective NRI on Self-Reported and Experimental Pain Measurements at Rest in Patients with Chronic WAD

The results regarding cognitive performance are reported elsewhere [22]. The results from the pain measurements before aerobic exercise in patients with chronic WAD were compared using a mixed model to analyze the influence of selective activated serotonergic or noradrenergic descending pathways on self-reported pain, PPTs, TS, CPM, and cuff pressure VNRS 3. The fixed factor of condition (citalopram, atomoxetine, or no-medication) did not have any significant effect on the results for PPTs, TS, and CPM at either test site (shoulder = local or calf = remote) (*p*-values ranging from 0.096 to 0.913, Table 2). In all but one outcome parameter (CPM at the shoulder; Supplementary Table S1), the occupational status of the participants was a significant confounder. Therefore, additional analyses taking into account the occupational status were conducted and reported below in the final paragraph of this Section 3 and in Supplementary Table S1.

### 3.3. The Isolated Effect of a Single Dose of a SSRI or a Selective NRI on Exercise-Induced Hypoalgesia in Patients with Chronic WAD

The self-reported and experimental pain measurements were analyzed over the three experimental conditions. Detailed results are reported in Table 3. No significant interaction effects (experimental condition x time) were found for any of the pain measurements. Equally for the fixed effect of experimental condition (no medication, citalopram, atomoxetine), no significant effects were found for any of the outcome measures. Only for CPM at the shoulder was a significant time effect found. Post hoc tests revealed positive CPM values post-exercise compared to negative CPM values pre-exercise, meaning an impaired CPM effect after aerobic exercise (i.e., exercise-induced hyperalgesia) in contrast to a normal CPM effect (hypoalgesia) before aerobic exercise (mean difference −0.394 (0.169 SE) *p* = 0.022). Other outcome measures did not show any significant time effect. 

### 3.4. Confounding Effect of Occupational Status on the Effect of a Single Dose of a SSRI or a Selective NRI on Exercise-Induced Hypoalgesia in Patients with Chronic WAD

Within all the best fitted mixed models, for six out of seven outcome parameters, the covariate occupational status appeared to have a significant influence (Supplementary Table S1). Post hoc analysis revealed a significant difference between the two most extreme subgroups, namely, participants who work full-time (*n* = 8) and participants who were unemployed (*n* = 9). “Unemployed” participants are considered to be part of the active working population but who are currently not at work; this group does not entail students or retired persons. Unemployed participants had their whiplash accident more recently with a median duration since the accident of 6 months (IQR 4 months) and a higher disability (median NDI 48, IQR 12) compared to the participants working full-time (median duration since accident: 48 months, IQR 38; median NDI 34, IQR 8.5; *p* < 0.05). Regarding the pain outcome measures in the unemployed group, the cuff pressure VNRS 3 showed a significant effect of the experimental condition. Bonferroni post hoc analysis showed that the citalopram condition had a lower cuff pressure VNRS 3 compared to the baseline condition (mean difference 21.3 (8.3 SE) *p* = 0.042), although it was not significant for the atomoxetine condition (mean difference 20.2 (8.7 SE) *p* = 0.077). Further, in the unemployed group, significant effects of time were found for TS and CPM at the shoulder. Bonferroni post hoc analysis showed a significantly higher TS (i.e., more pain facilitation) and higher CPM values (i.e., less hypoalgesia) after aerobic exercise compared to less pain facilitation and better hypoalgesia pre-aerobic exercise (TS: mean difference −0.394 (0.189 SE) *p* = 0.044; CPM: mean difference −0.458 (SE 0.214) *p* = 0.039). No significant condition effects were found in the subgroup of participants who had a full-time occupation. Besides a marginally significant interaction effect for TS at the calf (F = 2.988; *p* = 0.064), Bonferroni post hoc analysis of this interaction effect revealed an effect of time: reduced TS (i.e., less pain facilitation) after aerobic exercise compared to pre-exercise but only in the no medication condition (mean difference 0.938 (SE 0.397) *p* = 0.024). This interaction effect also contained significant results during the pre-exercise phase: a reduced TS (i.e., less pain facilitation) in the atomoxetine condition compared to the no medication (mean difference 1.063 (SE 0.397) *p* = 0.024) and citalopram conditions (mean difference 1.167 (SE 0.414) *p* = 0.034). Details regarding the sub-analyses and post hoc analyses can be found in Supplementary Table S1.

## 4. Discussion

This study aimed at examining the isolated effect of activating serotonergic or noradrenergic descending pathways on hypoalgesia at rest and in response to exercise in patients with chronic WAD. Contrary to our hypothesis, neither a single dose of SSRI nor selective NRI improved hypoalgesia at rest or in response to exercise in patients with chronic WAD. It was also found that a single dose of SSRI or selective NRI is unable to prevent post-exertional malaise following submaximal exercise in patients with chronic WAD. Taken together, a single dose of SSRI or selective NRI was unable to change hypoalgesia at rest, exercise-induced hypoalgesia, or post-exertional malaise in patients with chronic WAD. However, the occupational status of the patients with chronic WAD was identified as a confounder, as it had a consistent influence on the results. 

### 4.1. Neither a Single Dose of SSRI Nor Selective NRI Influence Hypoalgesia at Rest in Patients with Chronic WAD

The intake of one dose of atomoxetine or citalopram neither influenced the self-reported pain levels nor the experimental pain measurements in patients with chronic WAD. Serotonin and noradrenaline reuptake inhibitors have been proposed as one of the preferred pharmacological options for pain in patient with predominant nociplastic pain [5]. Theoretically, these monoamines have the potential to target some of the mechanisms underlying central sensitization. Impaired endogenous hypoalgesia as measured with the CPM paradigm is one of the characteristics of central sensitization. In humans, CPM is the psychophysical test paradigm aiming to measure descending inhibitory control mechanisms [41]. In animal work, this mechanism is called “diffuse noxious inhibitory control” (DNIC), a specific descending inhibitory control mechanism. Most results on the influence of monoamines on descending pain inhibitory mechanisms come from animal studies. In nerve-injured animals who experience a dysfunctional descending hypoalgesia, the DNIC can be restored through alteration of monoamine actions. More specifically, by blocking the serotonin descending facilitatory pathways or by enhancing noradrenaline action through the administration of NRIs, it is able to restore dysfunctional DNIC [42]. In humans, selective NRIs have been able to restore dysfunctional CPM in patients with chronic pain due to polyneuropathy [43]. It is suggested that a strong descending inhibitory system may protect against the development of chronic pain, and that the monoamine levels in the spinal cord are an important element of the analgesic effect of selective NRIs [2]. A dysfunctional descending inhibitory system is one of the mechanisms underlying central sensitization, and levels of serotonin as well as noradrenaline might influence this descending inhibitory system. Therefore, SSRIs and selective NRIs are expected to have a positive influence on the functioning of endogenous hypoalgesia in patients with chronic pain, including WAD. These expectations are partly met in some conditions, such as osteoarthritis, fibromyalgia, and low back pain, where selective NRIs have shown some effectiveness [44,45,46,47]. In contrast, the current study focused on the isolated effect of a SSRI and selective NRI, and this after a single dose. The assumption that altering the levels of serotonin or noradrenaline has an acute effect on clinical pain measurements including CPM in chronic WAD could not be confirmed. Possibly, this is due to the isolated effect compared to the combined effect of both actions. The combined effect of an SNRI is supposed to be more pronounced than the effect of SSRI and selective NRI alone in the treatment of chronic pain, and thus medications affecting both serotonin and noradrenaline might yield better results [4,48]. It would be interesting to study the effect of a single-dose SNRI on hypoalgia at rest and in response to exercise in patients with chronic WAD. 

### 4.2. Neither a Single Dose of SSRI Nor Selective NRI Influenced Exercise-Induced Hypoalgesia in Patients with Chronic WAD

The intake of one dose citalopram or atomoxetine in combination with an acute bout of submaximal aerobic exercise did not influence pain experience or experimental pain outcomes in patients with chronic WAD. These results do not support the hypothesis that an SSRI or selective NRI can improve exercise-induced hypoalgesia. Serotonin is involved in exercise-induced hypoalgesia in both humans and animals [16]. Exercise leads to an increase in serotonin concentrations in many brain areas, including those involved in pain control [17]. Still, studies combining serotonin, exercise, and pain are scarce. In patients with low back pain, spinal stabilization exercises resulted in the elevation of plasma serotonin levels, but these elevated plasma serotonin did not lead to reductions in pain response [49]. There has been another attempt to investigate the effect of a SSRI (citalopram intravenously) in combination with exercise on pain. This study was performed in healthy people and people with chronic fatigue syndrome and comorbid fibromyalgia. Unfortunately, the latter study had to be terminated early due to intense side effects in healthy people as well as in the patient group, and no meaningful interpretation of the results was possible [50]. In the current study, citalopram and atomoxetine were ingested orally. Some side effects were reported, which might have led to the dropout of one participant. Other participants reported no side effects after intake of a single dose of citalopram or atomoxetine. It is reassuring that the orally ingested dose of citalopram was well tolerated in our chronic WAD sample and that the side effects did not result in a barrier for the therapeutic use. 

Besides the serotonin system, the adrenergic system is also activated during exercise and may participate in exercise-induced hypoalgesia. With the release of hormones and neurotransmitters such as catecholamines, these catecholamines may modulate the nociceptive pathways, leading to reduced firing rates of excitable cells [16]. The studies investigating the involvement of this system in exercise-induced hypoalgesia are scarce, and all of them were performed on rodents. In rats, the noradrenergic involvement in exercise-induced hypoalgesia was present in both aerobic and resistance exercises [51]. In humans, results on the influence of the noradrenergic system in combination with exercise on pain are lacking. To our knowledge, the current study is the first to investigate the influence of the noradrenergic system on the exercise-induced pain response in a human pain population. For the interpretation, it is important to mention that our study population did not show hyperalgesia in response to exercise during the no medication condition compared to healthy participants (detailed results and discussion published elsewhere [23]). The intake of a single dose of SSRI or selective NRI was not able to facilitate exercise-induced hypoalgesia, but it might be useful in counteracting exercise-induced hyperalgesia. However, the latter is not detectable in the present study population. 

The bout of submaximal aerobic exercise did influence the pain experience in response to exercise in patients with chronic WAD. Patients demonstrated reduced local endogenous hypoalgesia (measured with the CPM paradigm) at the shoulder after exercise. However, SSRI or selective NRI intake did not influence this response. At the calf, CPM demonstrated hypoalgesia before as well as after exercise, with no differences between experimental conditions. Results of the no medication condition in comparison with healthy participants have been reported elsewhere [23]. In summary, the patients with chronic WAD demonstrated generalized hyperalgesia—as measured through PPTs—before exercise. However, compared to healthy participants, no deviating response to exercise was present. The current analysis on the combined results of the three conditions did not lead to different conclusions. There is conflicting evidence around this topic, where some studies find decreased PPTs [13] and others neither hyperalgesia nor hypoalgesia in patients with chronic WAD [18] in response to aerobic exercise. This supports the hypothesis that exercise-induced hyperalgesia is not a general trait among patients with chronic WAD. Possibly, only a subgroup of patients with chronic WAD demonstrates exercise-induced hyperalgesia. 

### 4.3. Occupational Status Influenced the Effect of a Single Dose of SSRI and Selective NRI on Hypoalgesia at Rest and in Response to Exercise in Patients with Chronic WAD

The two most extreme groups—patients who were working full-time versus unemployed patients—did experience an effect of atomoxetine and citalopram intake. Within the unemployed group, the intake of citalopram resulted in lower cuff pressure, indicating a lower pain tolerance level. This negative effect of the single dose of SSRI was not seen for the other outcome parameters. Patients with chronic WAD are likely to have nociplastic pain, making them more vulnerable to side effects (possibly related to the increased sensitivity of the central nervous system) [52]. Further, in this unemployed group, both CPM and TS worsened after exercise. This indicates a lack of hypoalgesia and increased pain facilitation after aerobic exercise, which might relate to low levels of physical activity in the unemployed group. This effect did not differ between the condition with or without medication intake. It is interesting to see that this group consists of patients who only experienced relative recently their whiplash injury, with a mean of six months since the trauma. On top of this, they reported higher levels of pain and disability. In contrast to this, patients with a full-time occupation presented positive responses to the intake of atomoxetine, namely, a lower TS at the calf before the exercise. However, the positive effect of exercise on pain facilitation, the reduction of TS at the calf, was only present when no medication was taken. The mean time since the whiplash trauma in this group was on average 4 years. 

Taken together, the results of the sub-analysis indicate that patients with chronic WAD who are unemployed demonstrate a worse reaction on exercise and intake of citalopram had a negative influence on their pain. Patients with a full-time occupation demonstrated some positive effects of exercise on pain measurements and the intake of a selective NRI had a positive effect when not combined with exercise. The disease duration on itself did not influence outcome measures, indicating that the occupational status is not a reflection of the stage of chronicity. These results should be interpreted with caution since this is a sub-analysis based on small groups. Still, the results are surprising since the medication intake was expected to be more effective in patients with a worse clinical picture compared to patients with milder symptoms. These findings underline the importance of subgroup identifications and strategies to develop individualized pharmacological treatment plans in patients with chronic pain [53] or patients with chronic WAD specifically [54]. 

### 4.4. Study Limitations and Strengths

The results should be seen with the recognition of some limitations. Firstly, the study aimed to investigate the short-term effect of a single dose of atomoxetine and citalopram. Nonetheless, this type of medication is usually taken for a longer period and different responses might be seen investigating the interaction with exercise in patients with a stable intake of these medications. SSRIs have at least two weeks of steady state administration to produce noticeable anti-depressant effects for depressive disorders or analgesic effects for neuropathic pain, while the effect onset of atomoxetine in adults is approximately 2 weeks. Secondly, the participants were blinded for whether they had taken citalopram or atomoxetine, but unfortunately it was not possible to blind them for the test day without medication. The intake of a placebo would have solved this, but this was omitted from the study protocol for ethical reasons. Third, temporal summation is typically elicited with application of stimuli <0.33 Hz, which was not achieved in this study. Fourth, a healthy control group would have been useful for comparison and to establish whether or not hypoalgesia at rest and exercise-induced hypoalgesia in patients with chronic WAD was (dys)functional.

This study also has several strengths. First, this is one of the only human experimental studies in patients with chronic pain that explores the potential role of various brain neurotransmitters in explaining dysfunctional pain modulation at rest and during exercise. The crossover experimental design is another major strength. The study design allows us to single out the effect of the various experimental conditions without between-subject variability. The used statistical methodology is also a strength of this study. The mixed models analysis that accounted for the many possible confounding factors allowed us to interpret the data with knowledge of its influencing factors and without data loss due to dropout. 

## 5. Conclusions

This study showed that a single dose of citalopram or atomoxetine was unable to improve hypoalgesia at rest, exercise-induced hypoalgesia, or post-exertional malaise in patients with chronic WAD. However, the occupational status of the patients with chronic WAD was identified as a confounder, with patients who were unemployed seeming to have more negative effects of exercise and experience the additional negative effect of citalopram, whereas patients with a full-time occupation experienced some benefit of exercise and might have additional positive effects of the intake of atomoxetine.

## Figures and Tables

**Table 1:** Demographic data and exercise characteristics in people with chronic WAD.

	Chronic WAD (*n* = 25)			
Age, years	40.7 (10.7)			
Sex, *n*	10 men (40%) 15 women (60%)			
Body mass, kg	73.8 (14.4)			
Height, cm	171.7 (8.8)			
BMI, kg/m^2^	24.9 (3.3)			
Disease duration, months	55.1 (84.2)			
Occupational status, *n*	9 inactive (36%) 6 part-time (24%) 8 full-time (32%); 2 student (8%) 0 retired (0%)			
Education level, *n*	0 primary education (0%) 9 secondary education (36%) 11 bachelor’s degree (44%) 3 master’s degree (12%)			
Antidepressants, *n*	1 (4%)			
Analgesics, *n*	8 (32%) ^a^			
Anti-epileptica, *n*	0 (0%)			
Legal conflict, *n*	13 no (52%); 1 employer (4%); 9 insurance company (36%); 1 employer and insurance company (4%)			
Successful blinding of patients	96%			
Successful blinding of assessors	100%			
	**No Medication** **(*n* = 25)**	**After Intake Citalopram** **(*n* = 20)**	**After Intake Atomoxetine** **(*n* = 22)**	***p* Value** ^b^
Neck Disability Index, /100	40.24 (34.97–45.51)	38.65 (33.25–44.05)	38.73 (33.38–44.08)	>0.05
Submaximal aerobic exercise characteristics				
Resting heart rate, beats per minute	82.96 ^c^ (77.78–88.14)	84.74 (79.05–90.42)	91.59 ^c^ (86.12–97.06)	<0.05 ^c^
Duration aerobic cycling exercise, minutes	4.80 (4.26–5.34)	5.12 ^c^ (4.55–5.69)	4.49 ^c^ (3.94–5.05)	<0.05 ^c^
Max Wattage	120 (106.54–133.46)	128.05 ^c^ (113.86–142.24)	112.30 ^c^ (98.41–126.18)	<0.05 ^c^

Values are presented as mean (SD) for data which were normally distributed or number (%) for categorical data. ^a^ All participants discontinued medication intake 2 weeks prior to study participation and on the test day (except for Citalopram or Atomoxetine according to randomization). ^b^ Statistical analyses were performed using random-intercept linear mixed models analysis; estimated means and 95% confidence intervals are presented. ^c^ There were significant differences between both indicated conditions. WAD = whiplash-associated disorders.

**Table 2:** Pain and pain modulation outcomes in people with chronic WAD in 3 conditions: baseline without medication intake, after intake of citalopram (SSRI), and after intake of atomoxetine (selective NRI).

Medication Condition	Estimated Marginal Means (SE)			
	Pre-Exercise		f-Score	*p*-Value
PPT at the shoulder ^ab^, kg/cm^2^				
No medication	1.767	(0.158)	0.814	0.450
Citalopram	1.898	(0.166)		
Atomoxetine	1.837	(0.162)		
TS at the shoulder ^b^, VNRS				
No medication	1.454	(0.328)	0.273	0.763
Citalopram	1.654	(0.355)		
Atomoxetine	1.431	(0.340)		
CPM at the shoulder ^b^, VNRS				
No medication	−0.460	(0.224)	1.024	0.368
Citalopram	−0.001	(0.250)		
Atomoxetine	−0.360	(0.238)		
PPT at the calf ^ab^, kg/cm^2^				
No medication	3.123	(0.342)	0.091	0.913
Citalopram	3.176	(0.356)		
Atomoxetine	3.088	(0.349)		
TS at the calf ^b^, VNRS				
No medication	1.798	(0.412)	1.304	0.283
Citalopram	1.761	(0.439)		
Atomoxetine	1.319	(0.424)		
CPM at the calf ^b^, VNRS				
No medication	0.738	(0.244)	0.383	0.683
Citalopram	0.707	(0.279)		
Atomoxetine	0.467	(0.258)		
Cuff pressure VNRS 3 ^b^				
No medication	88.67	(12.60)	2.480	0.096
Citalopram	71.77	(13.10)		
Atomoxetine	85.06	(12.82)		
VAS pain ^b^				
No medication	52.9	(4.1)	1.348	0.262
Citalopram	49.0	(4.3)		
Atomoxetine	49.6	(4.2)		

Statistical analyses were performed using random-intercept linear mixed models analysis; estimated means and standard error (SE) are presented. ^a^ Significant covariate pain medication, ^b^ significant covariate occupational status. PPT = pressure pain threshold, TS = temporal summation, CPM = conditioned pain modulation, VNRS = visual numerical rating scale, VAS = visual analogue scale, WAD = whiplash-associated disorders, no medication = baseline condition without medication intake. *n* = 25 for the no medication condition, *n* = 20 for the citalopram condition, *n* = 22 for the atomoxetine condition.

**Table 3:** Pain and pain modulation outcomes in people with chronic WAD in 3 conditions: baseline without medication intake, after intake of citalopram (SSRI), and after intake of atomoxetine (selective NRI); pre-, post-, and 24 h post-exercise.

Medication Condition	Estimated Marginal Means (SE)						Main Effect of Condition		Main Effect of Time		Interaction Effect		Bonferroni Post Hoc Test
	Pre-Exercise		Post-Exercise		24-h Post-Exercise		f-Score	*p*-Value	f-Score	*p*-Value	f-Score	*p*-Value	
PPT at the shoulder ^a^, kg/cm^2^													
No medication	1.675	(0.140)	1.657	(0.140)			2.005	0.14	0.165	0.685	0.486	0.616	
Citalopram	1.791	(0.145)	1.776	(0.145)									
Atomoxetine	1.734	(0.143)	1.834	(0.143)									
TS at the shoulder ^b^, VNRS													
No medication	1.384	(0.311)	1.584	(0.311)			0.187	0.829	0.016	0.900	0.995	0.373	
Citalopram	1.513	(0.330)	1.213	(0.330)									
Atomoxetine	1.350	(0.320)	1.509	(0.320)									
CPM at the shoulder ^b^, VNRS													
No medication	−0.460	(0.202)	0.100	(0.202)			0.898	0.410	5.408	0.022	0.405	0.668	Main effect of time Pre-exercise < Post-exercise mean difference −0.394 (SE 0.169) *p* = 0.022
Citalopram	−0.011	(0.226)	0.180	(0.220)									
Atomoxetine	−0.353	(0.215)	0.078	(0.215)									
PPT at the calf ^ab^, kg/cm^2^													
No medication	3.073	(0.342)	2.890	(0.342)			0.713	0.492	0.582	0.447	1.53	0.221	
Citalopram	3.107	(0.349)	2.924	(0.349)									
Atomoxetine	3.033	(0.345)	3.190	(0.345)									
TS at the calf ^b^, VNRS													
No medication	1.841	(0.408)	1.341	(0.408)			1.071	0.346	0.611	0.436	2.332	0.102	
Citalopram	1.833	(0.421)	1.708	(0.421)									
Atomoxetine	1.353	(0.414)	1.626	(0.414)									
CPM at the calf ^b^, VNRS													
No medication	0.668	(0.237)	0.548	(0.237)									
Citalopram	0.641	(0.264)	0.416	(0.264)			0.197	0.821	0.286	0.594	0.237	0.790	
Atomoxetine	0.445	(0.250)	0.514	(0.250)									
Cuff pressure VNRS 3 ^b^													
No medication	88.7	(12.6)	77.26	(12.6)									
Citalopram	72.4	(12.95)	74.35	(12.95)			2.262	0.109	0.795	0.375	0.896	0.412	
Atomoxetine	85.09	(12.77)	83.12	(12.77)									
VAS pain ^b^													
No medication	52.1	(4.8)	52.2	(4.8)	56.6	(4.8)							
Citalopram	47.4	(5.1)	47.2	(5.1)	52.6	(5.1)	1.347	0.263	1.644	0.196	0.616	0.652	
Atomoxetine	52.7	(4.9)	47.1	(4.9)	49.2	(4.9)							

Statistical analyses were performed using random-intercept linear mixed models analysis; estimated means and standard error (SE) are presented. ^a^ significant covariate pain medication, ^b^ significant covariate occupational status. PPT = pressure pain threshold, TS = temporal summation, CPM = conditioned pain modulation, VNRS = visual numerical rating scale, VAS = visual analogue scale, WAD = whiplash-associated disorders, No medication = baseline condition without medication intake. *n* = 25 for the no medication condition, *n* = 20 for the Citalopram condition, *n* = 22 for the Atomoxetine condition.

## Data Availability

Please contact the corresponding author for obtaining the research data. All requests for obtaining the research data will be considered by the research team.

## Supplementary Materials

The following supporting information can be downloaded at https://www.mdpi.com/article/10.3390/jcm12154977/s1, Supplementary Table S1: Pain and pain modulation outcomes in people with chronic WAD based on occupational status.
