# Supplementary material for: Unravelling Impaired Hypoalgesia at Rest and in Response to Exercise in Patients with Chronic Whiplash-Associated Disorders: Effects of a Single Administration of Selective Serotonin Reuptake Inhibitor versus Selective Norepinephrine Reuptake Inhibitor
Source: J Clin Med. 2023 Jul 28;12(15):4977. doi: 10.3390/jcm12154977 (PMC10419436; doi:10.3390/jcm12154977)
Supplement: Supplementary file 1 [file jcm-12-04977-s001.zip › jcm-2478493-supplementary.pdf]

Table S1: Pain and pain modulation outcomes in people with chronic WAD based on occupational status.

Medication condition	Estimated Marginal means (SE)					Main effect of condition		Main effect of time		Interaction effect		Bonferroni Post Hoc Tests
	Pre-exercise		Post-exercise		24-h post-exercise	f-score	*p*-value	f-score	*p*-value	f-score	*p*-value	
**PPT at the shoulder, kg/cm²**												
**Inactive**												
No medication	1.564	(0.148)	1.469	(0.148)		0.032	0.969	0.885	0.353	0.174	0.841	
Citalopram	1.543	(0.148)	1.458	(0.148)								
Atomoxetine	1.501	(0.151)	1.495	(0.151)								
**Working full-time**												
No medication	1.902	(0.255)	2.116	(0.255)		0.834	0.443	3.448	0.072	0.745	0.483	**Main effect of time** Pre-exercise < Post-exercise; mean difference −0.144 (0.077 SE) *p* = 0.072
Citalopram	2.111	(0.258)	2.118	(0.258)								
Atomoxetine	2.008	(0.255)	2.219	(0.255)								
**TS at the shoulder, VNRS**												
**Inactive**												
No medication	0.889	(0.488)	1.556	(0.488)		1.528	0.230	4.344	**0.044**	1.834	0.174	**Main effect of time** Pre-exercise < Post-exercise; mean difference −0.394 (0.189 SE) *p* = 0.044
Citalopram	0.889	(0.488)	0.778	(0.488)								
Atomoxetine	0.784	(0.486)	1.409	(0.486)								
**Working full-time**												
No medication	0.625	(0.436)	0.687	(0.436)		0.118	0.889	0.019	0.891	0.980	0.386	
Citalopram	0.398	(0.449)	0.683	(0.449)								
Atomoxetine	0.875	(0.436)	0.437	(0.436)								
**CPM at the shoulder, VNRS**												
**Inactive**												
No medication	−0.222	(0.292)	0.444	(0.292)		0.064	0.938	4.599	**0.039**	0.248	0.782	**Main effect of time** Pre-exercise < Post-exercise; mean difference −0.458 (0.214 SE) *p* = 0.039
Citalopram	−0.111	(0.292)	0.222	(0.292)								
Atomoxetine	−0.171	(0.308)	0.204	(0.308)								
**Working full-time**												

No medication	−0.312	(0.398)	-0.313	(0.398)		0.525	0.596	1.878	0.180	0.697	0.505	
Citalopram	−0.143	(0.425)	0.286	(0.425)								
Atomoxetine	−0.750	(0.398)	0.187	(0.398)								
**PPT at the calf kg/cm²**												
**Inactive**												
No medication	2.958	(0.564)	2.586	(0.564)		0.627	0.540	1.102	0.300	0.438	0.648	
Citalopram	2.685	(0.564)	2.538	(0.564)								
Atomoxetine	2.828	(0.569)	2.832	(0.569)								
**Working full-time**												
No medication	3.500	(0.351)	3.228	(0.351)		1.083	0.350	0.318	0.577	2.123	0.136	
Citalopram	3.306	(0.358)	3.414	(0.358)								
Atomoxetine	3.375	(0.351)	3.769	(0.351)								
**TS at the calf**												
**Inactive**												
No medication	1.222	(0.499)	0.667	(0.499)		0.668	0.518	0.045	0.834	1.616	0.212	
Citalopram	0.444	(0.499)	0.778	(0.499)								
Atomoxetine	0.566	(0.512)	0.941	(0.512)								
**Working full-time**												
No medication	1.563	(0.755)	0.625	(0.755)		2.587	0.090	2.936	0.096	2.988	0.064	**Interaction effect**<br>No medication<br>Pre-exercise > Post-exercise; mean difference 0.938 (SE 0.397) *p* = 0.024<br><br>Pre-exercise<br>No medication > Atomoxetine; mean difference 1.063 (SE 0.397) *p* = 0.024<br>Citalopram > Atomoxetine; mean difference 1.167 (SE 0.414) *p* = 0.034
Citalopram	1.667	(0.764)	1.025	(0.764)								
Atomoxetine	0.500	(0.755)	0.875	(0.755)								
**CPM at the calf ,VNRS**												

**Inactive**													
No medication	0.778	(0.277)	0.111	(0.277)			0.724	0.492	3.639	0.065	0.564	0.574	**Main effect of time** Pre-exercise > Post-exercise; mean difference 0.426 (SE 0.223) *p* = 0.065
Citalopram	0.222	(0.277)	0.111	(0.277)									
Atomoxetine	0.407	(0.294)	-0.093	(0.294)									
**Working full-time**													
No medication	0.125	(0.396)	0.125	(0.396)			0.078	0.925	0.341	0.562	1.361	0.268	
Citalopram	0.214	(0.424)	−0.143	(0.424)									
Atomoxetine	−0.500	(0.396)	0.438	(0.396)									
**Cuff pressure VNRS 3**													
**Inactive**													
No medication	80.1	(15.8)	57.0	(15.8)			4.080	0.025	1.223	0.276	1.954	0.156	**Main effect of condition** No medication > Citalopram mean difference 21.3 (SE 8.3) *p* = 0.042 Atomoxetine > Citalopram mean difference 20.2 (SE 8.7) *p* = 0.077
Citalopram	42.4	(15.8)	52.0	(15.8)									
Atomoxetine	72.0	(16.1)	62.7	(16.1)									
**Working full-time**													
No medication	133.0	(25.5)	121.5	(25.5)			0.084	0.920	0.000	0.985	0.713	0.498	
Citalopram	125.9	(25.8)	125.9	(25.8)									
Atomoxetine	123.3	(25.5)	136.3	(25.5)									
**VAS pain**													
**Inactive**													
No medication	69.78	(6.35)	74.56	(6.35)	70.11	(6.35)	0.927	0.401	0.171	0.844	0.599	0.644	
Citalopram	68.00	(6.35)	67.00	(6.35)	72.44	(6.35)							
Atomoxetine	68.91	(6.57)	62.35	(6.57)	67.79	(6.57)							
**Working full-time**													
No medication	27.69	(5.52)	14.94	(5.52)	17.25	(5.52)	0.015	0.986	2.559	0.087	0.572	0.684	**Main effect of time** Pre-exercise > Post-exercise mean difference 8,4 (SE 3,7) *p* = 0.085
Citalopram	22.57	(5.80)	18.14	(5.80)	17.71	(5.80)							
Atomoxetine	22.56	(5.52)	14.50	(5.52)	23.25	(5.52)							

Statistical analyses were performed using random-intercept linear mixed models analysis; estimated means and standard error (SE) are presented. PPT = pressure pain threshold, TS = temporal summation, CPM = conditioned pain modulation, VNRS = visual numerical rating scale, VAS = visual analogue scale, WAD = whiplash-associated disorders, No medication = baseline condition without medication intake. Inactive group: *n* = 9 for the no medication condition, *n* = 9 for the Citalopram condition, *n* = 8 for the Atomoxetine condition; Working full-time group *n* = 8 for the no medication condition, *n* = 7 for the Citalopram condition, *n* = 8 for the Atomoxetine condition.
